# The Dissector’s Manual of Practical and Surgical Anatomy

**Published:** 1857-03

**Authors:** 


ART. VI.—1. *The Dissector's Manual of Practical and Surgical Anatomy.* By ERASMUS WILSON, F. R. S., &c. Edited by WILLIAM HUNT, M.D., Demonstrator of Anatomy in the University of Pennsylvania. Third American (from the last London) edition; pp. 582. Blanchard & Lea: Philadelphia, 1856.

2. *The Practical Anatomist; or The Student's Guide in the Dissecting Room.* By J. M. ALLEN, M.D.; late Professor of Anatomy in the Medical Department of Pennsylvania College, &c.; pp. 631. Blanchard & Lea: Philadelphia, 1856.

3. *Practical Anatomy.* A new arrangement of the "London Dissector," with numerous modifications, additions, and illustrations. By D. HAYES AGNEW, M.D., Lecturer on Anatomy, &c.; pp. 310. J. B. Lippincott & Co.: Philadelphia, 1856.

PROBABLY no medical book requires so much judgment in the arrangement of its several subjects, and such simplicity and perspicuity of expression, as that which is intended as a guide to an individual just beginning the study of practical anatomy. No previous knowledge of the subject, excepting an acquaintance with the skeleton (without which, it is supposed, that no one would be likely to enter upon the work of dissecting), can be taken for granted; no allusions made to or mention of previously undescribed parts, or organs, without some immediate explanation. The majority of students, who enter the dissecting-room for the first time, are totally ignorant of many facts, of which one who has been long familiar with the study, can hardly conceive. The animal body is to them a sealed book, written in an unknown tongue; and he who engages to become their teacher, should possess that rare and peculiar talent, which, if so applied, would enable him to comprehend and satisfy the wants of an inquisitive child in reference to a watch, or some other such complicated piece of mechanism, which he may have undertaken to separate and study. He should have a perfectly distinct recollection not only of the generally recognized, but of the many unexpressed difficulties, which lay in his own way when, with scalpel in hand, he first essayed to discover the mysteries concealed beneath the natural covering of the human form divine; and what Peter Parley has done for the infant student of geography and history, he should be able to do for the relatively not less well-informed student of anatomy. Nay, more; for, while the comprehension of the child can grasp only the more evident and striking truths of geographical science, the student of medicine is supposed to be able to appropriate to himself the knowledge, not only of the attachments of a few of the larger muscles, and the course of some of the more important bloodvessels, but all those complex relations of parts, an acquaintance with which is absolutely necessary to the conscientious practitioner of medicine and surgery. Here, then, lies the difficulty, to combine completeness with simplicity; and, we are compelled to state that, so far as our acquaintance with anatomical literature goes, no writer on practical anatomy has yet come fully up, or rather down, to the standard. Of the three volumes, whose titles stand at the head of this notice, all aiming at the point in question, that of Mr. Wilson has been for some time in the hands of the profession and students of this country, and has received the stamp of general approbation, having now reached its third edition. Its excellencies are numerous, and, we dare say, without fear of contradiction from proper authority, that no American or foreign book, published in this country, comes so near to answering all the requirements of those to whom it is addressed as the original edition of the work, rearranged by Dr. Goddard, of this city. In the present edition, Dr. Goddard's arrangement is still preserved; but, like many other good books to which we might point, the good qualities have become somewhat obscured, and even in many places completely destroyed by the numerous additions which the work has received. We venture to predict that, unless the author or editor (for it is difficult to say who the responsible person is) should pursue an opposite course, substituting the scissors for the pen, that, by the time the book reaches its fourth or fifth edition, it will have attained a development leading to serious doubts as to whether it is intended for the novice or the experienced dissector, possessing neither the simplicity requisite for the former, nor the completeness wanted by the latter.

We could designate many parts that might with benefit be retrenched or expunged, but there is one which has always appeared to us as peculiarly out of due proportion, and we have been astonished that the work in passing through such experienced hands as Dr. Goddard and its present editor, should be still marked by the same inequality. We refer to the long account of the microscopic structure of the liver, which is made to occupy not less than six closely-printed pages. If there be a necessity for the introduction of so much matter of this sort in connection with one gland, which we cannot admit, the *practical* nature of so much of this kind of matter being a disputed point in books of more pretension, the same will hold true in regard to a number of other organs, the brain for example, the minute structure of which is not even alluded to in an intelligible manner.

The work of Dr. Allen, in point of size and appearance, bears a close resemblance to that of Mr. Wilson, but is inferior to it in convenience of arrangement and perspicuity of description. It is, however, an excellent book in many respects, and probably has the advantage of the former in its illustrations, which are more numerous, better selected, and better printed, although, in reference to this last-mentioned particular, neither has any reason to boast. Indeed, we cannot let the opportunity pass of advising the publishers, when they get out another edition of Mr. Wilson's book, to provide themselves with a new set of wood-cuts; many of those used in the present issue, and also in Dr. Allen's book, are evidently so much worn as to entitle them to an honorable pension, or, what would probably be more humane, to a complete annihilation by fire. If Dr. Allen will excuse us, however, we think we can put him in the way of improving his Dissector when a second edition shall be called for. His great fault is evidently a want of definiteness, and, more especially, too much presumption upon the student's previous knowledge of the subjects discussed. For example, the book opens with an account of the anatomy of the face, which, after stating its general limits, the author says it is convenient to consider as divided into several regions; "as the parotid, the masseteric, the buccal, the mental, the labial, the nasal, the orbital, and the malar. The location of each of these divisions is indicated by its name. It is not necessary for our present purpose to define their boundaries." Surely, in penning this paragraph, the author must have forgotten that he was addressing himself to students who cannot be expected to understand the significance of such terms as masseteric, buccal, &c., much less know the boundaries of the regions to which they are applied. We, therefore, beg leave to differ with him upon the assertion contained in the last sentence of the quotation, and take the liberty of saying that, if it was proper to mention the names of these regions in this connection, it was absolutely necessary that the student should be informed where they are situated, and what their boundaries are. We supposed upon first reading this paragraph, that the author only intended to defer the information until proceeding to an account of each region separately, but on looking a little further in the same section, we found that he at once enters into an account of the structures contained in each, without so much as an allusion to their situation or limits.

Again, in the third paragraph of this same section, we read, in reference to the parotid and masseteric regions, that, "as the integument is raised and reflected forwards, the platysma myoides will be found traversing the anterior part of these regions." Now, aside from a general want of clearness in regard to the precise position and direction of the platysma myoides in this situation, the author does not once tell the student that this big name, which it is reasonable to suppose the latter has never seen or heard before, is applied to a muscle. It is true that by looking in his dictionary he would find the term *myoid* to mean, like a muscle, but unless he should happen to find there also an account of the muscle in question, we doubt whether he would be much the wiser for his trouble.

We find this same want of clearness, and these same omissions running throughout the entire work. Thus, in another part of the same section, it is stated that "the buccinator muscle is separated behind from the ramus of the inferior maxilla and masseter by a mass of fat and two of the *buccal glands* called molar." No account of these glands having been previously given, and no word of intimation as to their nature, size, or appearance, accompanying the mention of their names, it appears to us that a *new* student would be sorely puzzled to know what is meant; and here, too, his dictionary would avail him but little; for from it he would simply learn that the term buccal had something to do with blowing, and molar with grinding, neither one of which definitions would be likely to satisfy his wants. Let it be borne in mind, that it is not to the use of these terms that we object, but if the author thought fit to mention the existence of the organs to which they are applied, he should have furnished the uninitiated student with some intimation of their nature, size, or appearance.

Section II begins with some directions for dissecting the orbital region, in which connection the student is told, that the "integument in this region should be removed by cutting in the direction of the fibres of the orbicularis," but what this direction is, he is not informed, nor is there any reference to a wood-cut by which he might be enlightened. In a subsequent paragraph, we are told that this same orbicularis originates from the *tendo-palpebrarum*, but there is not in this connection, nor in any previous part of the book, the least clue as to the situation, size, or attachment of this important structure.

We hope that Dr. Allen will see the justice of these criticisms, and numberless others of a similar kind might be made, for however small the faults alluded to may appear to an individual as familiar with the subject as he doubtless is, to one who, like the writer, has but very recently experienced the difficulties of a first dissection, they are of very serious import.

The "London Dissector" has never been a very great favorite with the anatomical students of this country, but possesses merits far beyond its previous lack of success. Dr. Agnew's rearrangement has very much improved the working qualities of the book; and with the benefit of numerous illustrations which in this edition have for the first time been introduced, to say nothing of the excellent manner in which the publishers have performed their part, we look forward to its speedy adoption by many of the institutions of this country. We are a little surprised, however, to find that a teacher of Dr. Agnew's keen anatomical eyes should have allowed such a plate as No. 53 to appear as an illustration of the parts intended. The figure in question we have always considered as one of the most serious blots upon the work from which it was originally taken, and we certainly never expected to see it repeated.

